# Editorial: Waterfowl production and management strategies: nutrition, genetics and breeding, and diseases prevention

**DOI:** 10.3389/fvets.2023.1352086

**Published:** 2024-01-05

**Authors:** Zhiming Zhu, Dan Liu, Chunhe Wan

**Affiliations:** ^1^Institute of Animal Husbandry and Veterinary Medicine, Fujian Key Laboratory of Animal Genetics and Breeding, Fujian Academy of Agricultural Sciences, Fuzhou, China; ^2^Institute of Animal Husbandry and Veterinary Medicine, Fujian Key Laboratory for Avian Diseases Control and Prevention, Fujian Academy of Agricultural Sciences, Fuzhou, China; ^3^China Institute of Veterinary Drug Control, Beijing, China

**Keywords:** waterfowl production, management strategies, nutrition, genetics and breeding, diseases prevention

China has a long history of waterfowl breeding and is the world's earliest domestication of waterfowl in the world. At the same time, it is also the world's largest country of waterfowl breeding and consumption. The waterfowl industry in China includes three major industries: meat ducks, egg ducks, and geese. The waterfowl industry is not only a characteristic industry in China but also an important part of animal husbandry, which plays an important role in meeting the effective supply and diversified market demand of livestock and poultry products. However, in recent years, China's waterfowl industry as a whole has declined (1), and the development of the waterfowl industry has faced many difficulties and enormous challenges, such as outdated technology for waterfowl feed configuration, which usually refers to the nutritional standards of chickens. The breeding technology is relatively backwards, the key breeding techniques have not been breakthrough for a long time, and the breeding efficiency is low. The overall situation of epidemic diseases still presents a negative trend of “old diseases remain unresolved and new diseases constantly emerge.” In the past few decades, researchers have paid special attention to the nutrition, genetic breeding, and disease prevention and control of waterfowl.

In recent years, nutrition research on waterfowl has mainly focused on the study of nutritive or non-nutritive additives, feed resource development, and nutritive value assessment. Genetic breeding research on waterfowl has mainly focused on the genetic basis of important economic traits, conservation and innovative utilization of germplasm resources, production management methods and breeding technology methods of waterfowl breeding.

This Research Topic aimed to report on the latest research progress in the nutrition and genetic breeding of waterfowl. There are 4 articles on this issue, which are about the effect of nonconventional feed on the performance of waterfowl and the research of molecular markers related to waterfowl. The Research Topic of this article focuses on the field of waterfowl nutrition and genetic breeding, evidenced below by reference to the designated area's letters.

## 1 Research and analysis of unconventional feed on the production performance of waterfowl

This thematic section contains three articles on the study of the production performance of waterfowl fed different feeds. They relied on experimental results focusing on geese and laying ducks, with 2 articles studying geese and 1 article studying ducks.

With the rapid development of large-scale and intensive livestock farming, livestock diseases have become increasingly complex and diverse, and livestock intestinal health problems have become increasingly prominent. The healthy development of animal husbandry has thus necessitated a search for alternative safe, efficient, and reasonable “green feed.” The development and utilization of unconventional feed resources is an important way to alleviate the shortage of conventional energy and protein feed resources, reduce feeding costs and improve economic benefits. Here, Wang et al. studied the effects of feeding whole-plant ensiled corn stover on the growth performance, blood parameters, and cecal microbiota of Holdobagy geese. Their study suggested that whole-plant ensiled corn stalks (WECS) could be a long-term stable feed source for geese, which could contribute to reducing feeding costs. However, it was important to monitor the amount of WECS added, as it could affect the absorption of Zn by geese. Supplementation of Zn in the diet might be necessary to meet the needs of geese. Notably, adding 30% WECS to the diet could increase the richness, evenness, and diversity of the cecal microbiota, indicating potential benefits to gut health. These findings contribute to optimizing goose farming practices, improving feed utilization, and enhancing the overall productivity and wellbeing of geese. Fu et al. investigated the effects of adding 2.5%, 5.0%, and 7.5% fermented feed to the basic diet on the growth performance, serum biochemical indexes, antioxidant capacity, and intestinal health of lion-head goslings. Their research indicated that dietary fermented feed (FF) supplementation improved the growth performance, serum biochemical indexes, antioxidant capacity and intestinal flora structure of lion head geese. Notably, dietary 7.5% FF supplementation was optimal for the growth and intestinal health of lion-headed geese.

In a study of unconventional feed for ducks, Chen et al. found that dietary supplementation with honeycomb extracts positively improved the egg nutritional and flavor quality and serum antioxidant and immune functions of laying ducks. Their research results showed that compared with the control group, honeycomb extract addition significantly increased the average daily feed intake but did not affect the other laying performance indexes, egg quality or serum biochemical indexes of laying ducks. Dietary supplementation with honeycomb extracts significantly increased crude protein content and decreased the contents of cholesterol and trimethylamine in eggs. Diets supplemented with 1.5 g/kg honeycomb extracts significantly improved egg total amino acid and flavor amino acid contents, monounsaturated fatty acid and polyunsaturated fatty acid composition and enhanced the serum antioxidant activity and immune functions of ducks.

## 2 Studies on molecular markers related to waterfowl

This thematic section contains one article on the study of differentially expressed ovarian genes and signaling pathways in Gaoyou ducks. Poultry breeders usually select the ovary to study egg-laying traits and identify differences related to these traits at the histological and molecular levels to explore the regulatory mechanism of egg-laying performance. Gaoyou duck is famous in China and abroad for its good production of double-yolk eggs. However, there has been no systematic research on the egg-laying characteristics of the Gaoyou duck, which limits the development and utilization of breed resources. To identify essential genes related to ovarian development, Zhang et al. constructed transcriptome profiles of Gaoyou duck ovaries at different physiological stages by RNA-Seq and GO (performed gene ontology) and KEGG (Kyoto Encyclopedia of Genes and Genomes) analyses on differentially expressed genes (DEGs). Their results suggested that eight candidate signaling pathways were critical for ovarian development, including MAPK signaling, progesterone-mediated oocyte maturation, cell adhesion molecule, NOD-like receptor signaling, ECM-receptor interactions, focal adhesion, TGF-β signaling and phagosome. Finally, five key DEGs were identified to participate in ovarian development, including *TGIF1, TGFBR2, RAF1, PTK2*, and *FGF10*. The findings of this study revealed the mechanisms underlying the molecular regulation of related genes in Gaoyou duck ovarian development and provided a basis for further understanding egg production in Gaoyou ducks, particularly double yolk eggs.

## 3 Perspectives

In conclusion, this Research Topic provided relevant research on the nutrition and genetic breeding of waterfowl, with a focus on the impact of unconventional feed on the production performance of waterfowl and the identification of candidate genes and signaling pathways related to egg duck ovaries. These studies have certain reference value for future water poultry nutrition, genetic breeding, and disease prevention and control. The feeding standards for waterfowl are mostly based on the USA NRC (1994), former Soviet Union standards, or chicken standards (2), and there is an urgent need to develop nutritional requirements standards suitable for ducks and geese. The application of molecular breeding in waterfowl is still in the exploratory stage, and how to use molecular breeding technology to accelerate the progress of waterfowl breeding is an important studied and explored direction. Then, the following aspects are provided as references for nutrition, genetics and breeding, and diseases prevention.

(1) Continuously improving the research on nutrient requirements for waterfowl, utilizing ideal amino acid patterns based on nutritional needs, researching and developing low-protein diets to reduce environmental nitrogen emissions. Suitable feeding standards should be formulated scientifically and reasonably, and a nutritive value database of waterfowl feed should be established.

(2) Molecular breeding technology is currently the most important method and means of genetic breeding for waterfowl. Understanding the molecular genetic basis of traits such as growth, meat quality, and egg production in waterfowl, identifying relevant major or candidate genes, and screening effective molecular markers is beneficial for improving breed accuracy and accelerating the progress of waterfowl breeding. In this field, the comprehensive use of various information, such as multiomics information, comparative genome information, gene expression information, and regulatory information, will help more accurately screen important molecular markers.

## Author contributions

ZZ: Writing—original draft. DL: Resources, Supervision, Writing—review & editing. CW: Supervision, Writing—review & editing.
